# Retinal Muller Glia Initiate Innate Response to Infectious Stimuli via Toll-Like Receptor Signaling

**DOI:** 10.1371/journal.pone.0029830

**Published:** 2012-01-09

**Authors:** Ashok Kumar, Nazeem Shamsuddin

**Affiliations:** 1 Department of Ophthalmology, Kresge Eye Institute, Wayne State University, Detroit, Michigan, United States of America; 2 Department of Anatomy and Cell Biology, Wayne State University, Detroit, Michigan, United States of America; McMaster University, Canada

## Abstract

Ocular surgeries and trauma predispose the eye to develop infectious endophthalmitis, which often leads to vision loss. The mechanisms of initiation of innate defense in this disease are not well understood but are presumed to involve retinal glial cells. We hypothesize that retinal Muller glia can recognize and respond to invading pathogens via TLRs, which are key regulators of the innate immune system. Using the mouse retinal sections, human retinal Muller cell line (MIO-M1), and primary mouse retinal Muller cells, we show that they express known human TLR1-10, adaptor molecules MyD88, TRIF, TRAM, and TRAF6, and co-receptors MD2 and CD14. Consistent with the gene expression, protein levels were also detected for the TLRs. Moreover, stimulation of the Muller glia with TLR 2, 3, 4, 5, 7 and 9 agonists resulted in an increased TLR expression as assayed by Western blot and flow cytometry. Furthermore, TLR agonists or live pathogen (*S. aureus*, *P. aeruginosa*, & *C. albicans*)-challenged Muller glia produced significantly higher levels of inflammatory mediators (TNF-α, IL-1β, IL-6 and IL-8), concomitantly with the activation of NF-κB, p38 and Erk signaling. This data suggests that Muller glia directly contributes to retinal innate defense by recognizing microbial patterns under infectious conditions; such as those in endophthalmitis.

## Introduction

Glial cells are found throughout the central nervous system (CNS). In the mammalian retina, three types of glia have been identified: Muller glia, astroglia or astrocytes, and microglia. Muller cells are the predominant glial cell type in the retina and have a unique anatomy with processes that span the entire retinal thickness, forming the retinal margins at the internal (ILM) and outer (OLM) limiting membranes retina [1], [2]. They surround neuronal cell bodies and processes within the retina and perform a wide range of functions, including structural stabilization, regulation of ion homeostasis, neurotransmitter recycling, and neuronal survival [2], [3], [4], [5]. Thus, the primary role of Muller glia is to maintain retinal neuronal health [6], [7]. However, when retinal homeostasis is perturbed as a result of trauma, neurodegeneration, inflammatory disease, or excitotoxicity, Muller glia becomes activated and undergo reactive gliosis [6]. The characteristics of Muller gliosis include cellular hypertrophy, upregulation of glial fibrillary acidic protein (GFAP) and the intermediate filament, vimentin [7]. Although virtually every retinal disease is associated with a reactive Muller gliosis, little is known about Muller glial innate response in infectious diseases of the retina. Considering their strategic location and predominance, it may therefore be postulated that Muller glia are more likely to encounter microbes than other cells. Thus, we hypothesized that Muller cells play an important role in defending the retina from infectious insults via the action of Toll-like receptors (TLRs).

Toll-like receptors (TLRs) are a family of evolutionarily conserved pattern recognition receptors that recognize microbial-associated molecular patterns (MAMPs) from diverse organisms, including bacteria, viruses, fungi, and parasites [8]. Currently, 13 TLRs have been identified: TLRs 1–9 are common to mouse and human, TLR 10 is only found in humans, and TLRs 11–13 are unique to the mouse [9]. Activation of TLRs on immune cells by pathogens or their products initiate the innate response characterized by the expression of proinflammatory mediators and anti-microbial effector molecules [10]. TLRs also recognize endogenous ligands termed as damage-associated molecular patterns (DAMPs), and mediate the host's inflammatory response to injury and stress [11]. The initial inflammatory responses mediated by TLRs are required for host defense against invading pathogens and tissue repair. However, exaggerated or chronic inflammation induced by activation of these receptors may also lead to tissue damage and development of autoimmune disorders [12].

Several studies have demonstrated the expression of TLRs in various cell types in the CNS, including microglia, astrocytes, neurons, and vascular endothelial cells [13]. Accumulating evidence supports that TLRs play a major role in brain infection and injury, and are involved in autoimmune neuropathy and neurodegenerative disorders [12], [14], [15], [16]. Since, the retina is a part of CNS, it is logical to propose that similar to the brain, retinal glial cells also express TLRs. Although, few recent studies from our [17], [18] and other laboratories have documented the expression of TLRs in the retina [19], [20], [21], relatively little is known about the role of TLR signaling, particularly in infectious endophthalmitis. Moreover, to our knowledge, a full characterization of TLR expression and responsiveness to TLR ligands in retinal Muller glia has not previously been carried out. In the present study, we have investigated the recognition and initiation of Muller glial innate response by TLR ligands and live pathogens. Our data demonstrates the expression of all TLRs by Muller glia, which enables the detection of both Gram-positive, Gram-negative bacteria and fungal pathogens. These findings emphasize the potentially important role the Muller glia plays in regulating retinal innate defense.

## Results

### Retinal Muller glia express Toll-like receptors

**Figure 1:**
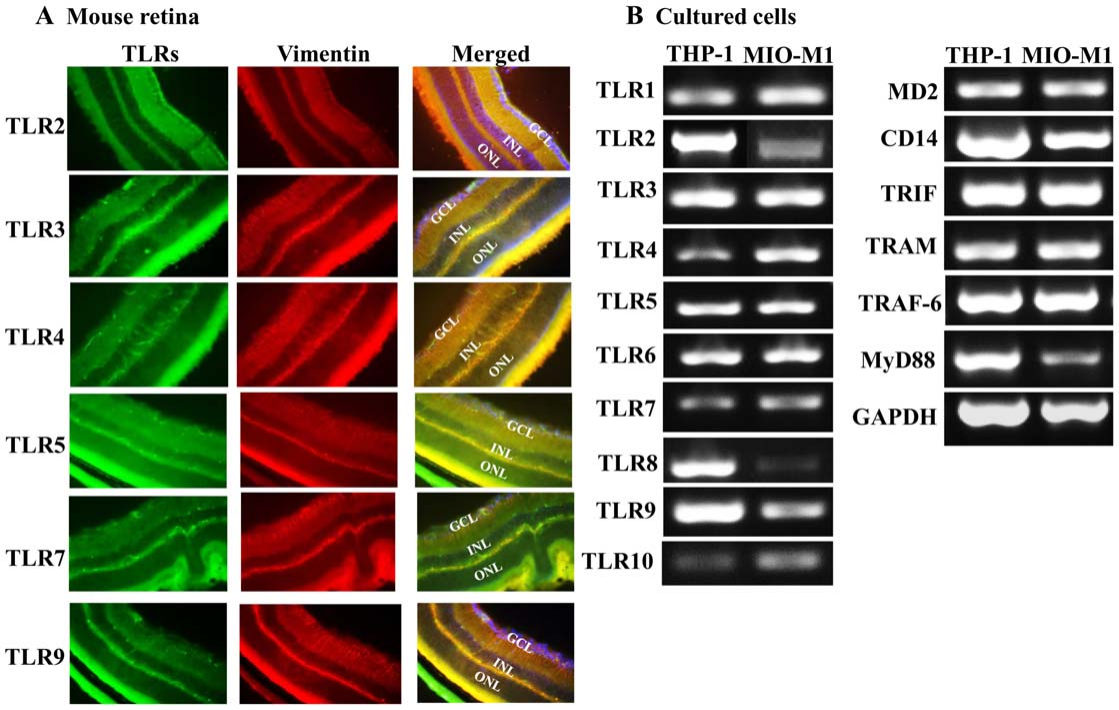
In vivo and in vitro expression of TLRs in retinal Muller glia. (**A**) Eyes from C57BL/6 mice were enucleated and embedded in OCT, and cryo-sections were stained with indicated TLR antibodies. Double immunofluorescence labeling was performed using anti-TLR 2, 3, 4, 5, 7 and 9 (green) antibodies and anti-vimentin (red) antibody, a marker for Muller glia cells. TLRs expression co-localizes to Muller glia, as the Muller cell bodies and radial running processes were immunopositive. (**B**) To assess the expression of TLRs, other co-receptors, and adaptor molecules, RNA extraction followed by RT-PCR was performed on cultured human retinal Muller glia cell line (MIO-M1) and human monocyte cell line (THP-1), as a positive control. Results shown are representative of two experiments with similar results. GCL, ganglion cell layer; INL, inner nuclear layer; ONL, outer nuclear layer. Magnification 20×.

TLRs are known to play a key role in initiation of early innate responses [22]. We hypothesized that in the case of endophthalmitis, retinal Muller glia recognize infectious stimuli via TLR signaling and focused our attention assessing their role in Muller glial innate response. First, we performed an immunohistochemical analysis to determine the cellular localization of TLRs in the mouse retina. Double immunofluorescence labeling of the retina sections using specific antibodies to key TLRs (TLR 2, 3, 4, 5, 7 and 9) as well as the cell markers for the Muller cells revealed that all of the six TLRs studied co-localize with radially running vimentin positive cells, an indication of Muller glia (Fig. 1A). To confirm the expression of TLRs specifically in Muller glia, we performed RT-PCR analysis on the spontaneously immortalized human retinal Muller glia cells line, MIO-M1 [23]. As shown in Figure 1B, MIO-M1 cells express all human TLRs (TLR1 to 10); additionally, they were also found to express co-receptors (MD2 and CD14) and adaptor molecules (TRIF, TRAM, TRAF-6, and MyD88). All amplified products were of predicted size for their respective genes as is evident by running parallel PCR with cDNA from a human monocytic cell line (THP-1), which is known to express all TLRs [24]. Thus, we conclude that retinal Muller glia express TLRs.

### Stimulated retinal Muller glia exhibited increased TLR expression levels

**Figure 2:**
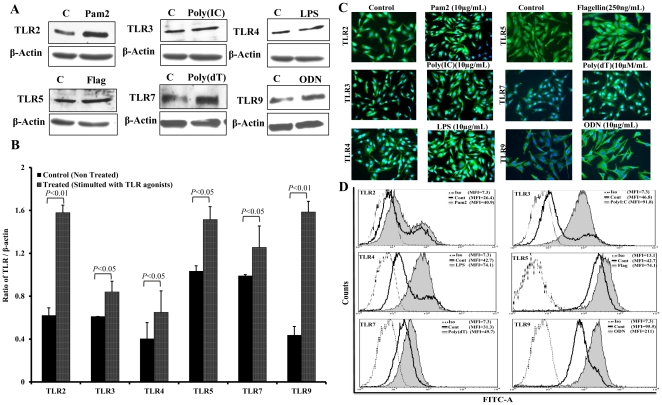
Induced TLR expression in Muller glia challenged with TLR ligands. MIO-M1 cells were left untreated (C) or stimulated with indicated TLR ligands Pam2Cys (10 µg/ml), Poly(IC) (10 µg/ml), LPS (10 µg/ml), Flagellin (250 ng/ml), Poly(dT) (10 µM/ml), and ODN (10 µg/ml) for 4 h. Cells were lysed for Western blot analysis using specific anti-TLR and β-actin (control) antibodies (**A**). The band intensity was quantified by densitometric analysis and presented as relative band intensity of TLR vs β-actin (**B**). Induced expression of TLRs was further confirmed by immunostaining using indicated anti-TLR antibodies (**C**) and for quantitation, cells were stained for FACS analysis and presented as mean fluorescent intensity (MFI) of respective TLR positive cells (**D**). The data shown are representative of duplicate experiments. The indicated p values (*t*-test) are comparisons of unstimulated vs TLR agonist stimulated cells. Magnification 20×.

The previous results indicated that retinal Muller glia express TLRs at basal levels. To determine whether their expression is altered following stimulation, MIO-M-1 cells were challenged with TLR ligands, and their respective TLR expression was assessed by Western blot, immunohistochemistry, and flow cytometry analysis. Clearly, stimulation of MIO-M1 cells by indicated TLR ligands for 4 h up-regulated the protein levels of TLR2, 3, 4, 5, 7 and 9 (Fig. 2A). The semi-quantitative analysis by measuring band intensities revealed dramatic increases in levels of TLR2 and 9 compared to others (Fig. 2B). In order to ascertain the induced expression of TLRs, we performed immunohistochemistry of stimulated Muller glia using anti TLR antibodies. Consistently, our immunofluorescence data also showed the expression and upregulation of various TLRs in Muller glia (Fig. 2C). Since immunofluorescence is not a quantitative method, we performed flow cytometry analysis. To this end, our data showed induced TLR expression, and marked increases in MFI values were observed for TLR3, 4 and 9 (Fig. 2D). Taken together, these results suggest that infectious stimuli elicit TLR expression in retinal Muller glia.

### TLR ligands induced the activation of p38 and Erk MAPKs in retinal Muller glia

**Figure 3:**
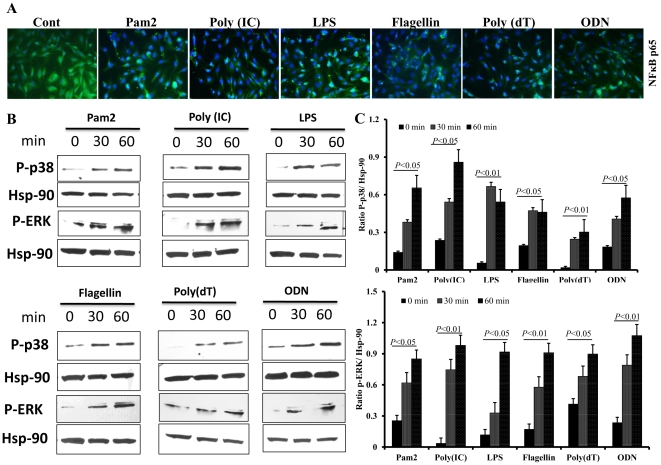
TLR agonist stimulation activated NF-κB, p38 and ERK signaling in Muller glia. To detect the nuclear translocation of NF-κB p65, MIO-M1cells were cultured for 60 min in the presence of indicated TLR ligands. Cells were then fixed, permeabilized, and immunostained with Ab against p65 subunit of NF- κB (**A**). To assess the activation of p38 and ERK signaling, following TLR ligand stimulation for 30 and 60 min, MIO-M1cells were lysed for Western blot analysis using antibodies against phospho-p38 (P-p38) and phospho-ERK (P-ERK). Antibodies against non-phosphorylated p38 and ERK were used to detect their total levels, and Hsp-90 was used as an equal protein loading control (**B**). The band intensity was quantified by densitometric analysis and presented as relative band intensity of TLR vs Hsp-90 (**C**). The indicated p values (one-way ANOVA) are comparisons of stimulated cells at 0 min vs 30 and 60 min. MIO-M1 cells challenged with TLR ligands showed time-dependent manner activation of p-38 and p-ERK signaling pathways. The data shown are representative of duplicate experiments.

The induced expression of different TLRs following stimulation suggests that TLR downstream signaling might also be activated in Muller glia. To test this, MIO-M1 cells were challenged with ligands for TLR2, 3, 4, 5, 7 and 9, and the activation of p38 and Erk MAPKs was assessed by Western blot. All TLR agonists induced the activation of p38 and Erk, as evidenced by increased levels of phosphorylated p38 and Erk (Fig. 3A). The semi-quantitative analysis showed that although increased phosphorylation of p38 and Erk were detected at 30 min, the maximum level of phosphorylation was observed at 60 min post stimulation (Fig. 3B). Together, these findings demonstrate that TLR ligand stimulation induces p38 and Erk signaling in Muller glia, an indication of their functional TLRs.

### Muller glia produces inflammatory mediators in response to TLR ligand and pathogen challenge

**Figure 4:**
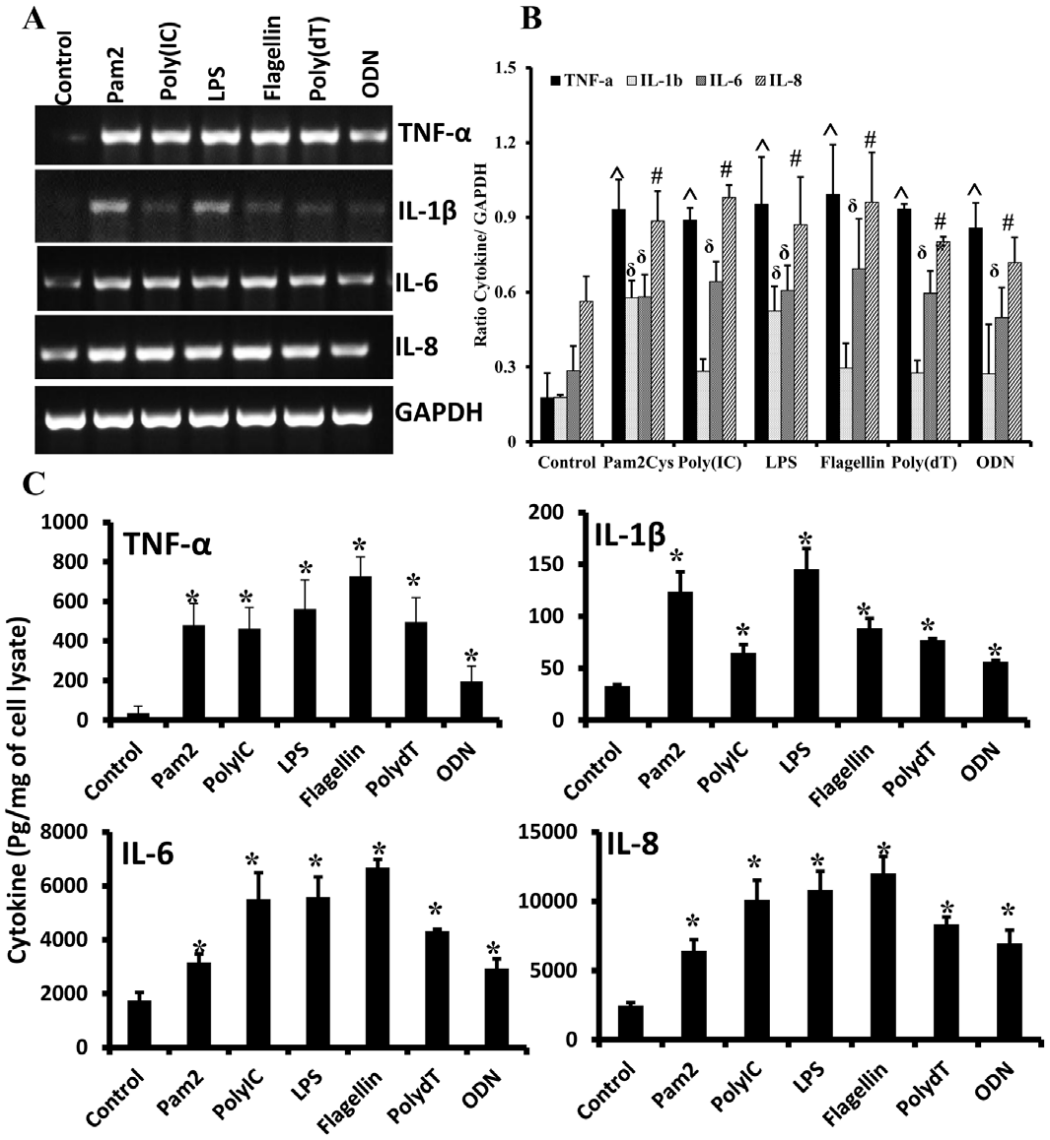
TLR agonists induced expression and secretion of inflammatory mediators in Muller glia. MIO-M1 cells were stimulated with indicated TLR ligands. After 4 h, total RNA was extracted, reverse transcribed, and amplified using specific primers (Table 1), with GAPDH as the control. PCR products were separated by electrophoresis and stained with ethidium bromide (**A**). The band intensities of PCR products were quantified by densitometric analysis and presented as relative band intensity of cytokines vs GAPDH. (**B**). In another experiment, MIO-M1cells were challenged with TLR agonists for 8 h and secretion of indicated cytokine/chemokine were measured by ELISA (**C**). The amount of cytokines was normalized with protein concentration of cell lysate (picograms per milligram cell lysate). One-way ANOVA was performed for statistical analysis of induced expression of TNF-α (∧*p*<0.01), IL-1β (^δ^ *p*<0.05), IL-6(^δ^ *p*<0.05), and IL-8 (^#^ *p*<0.05) in control vs various TLR agonist stimulated cells. Similar analysis was performed for ELISA data (*p<0.05). The data shown are cumulative of three experiments performed in duplicate.

**Figure 5:**
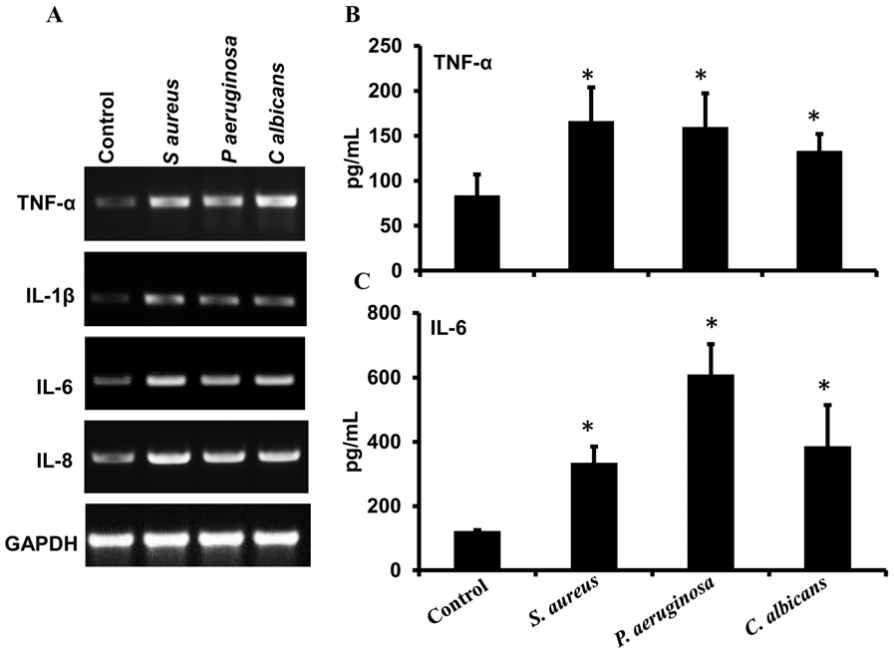
Muller glia expresses and secretes proinflammatory mediators in response to live pathogen challenge. MIO-M1 cells were stimulated with *S. aureus* (Gram-positive bacteria), *P. aeruginosa* (Gram-negative bacteria), and *C. albicans* (fungi) for 4 h. At the end of the incubation period, cells were used for RNA extraction followed by RT-PCR (**A**), whereas the culture supernatant was used for measurement of TNF-α (**B**), and IL-6 (**C**). The indicated statistical differences are comparisons of unstimulated (control) vs TLR agonist stimulated cells: *p<0.05 (one-way ANOVA).

To assess the biological relevance of induced p38 and Erk signaling and to further demonstrate the Muller glial responsiveness to different TLR ligands, we determined the effects of various TLR ligands on the expression of proinflammatory cytokines using RT-PCR. Compared to unstimulated controls, all TLR agonists induced the expression of IL-6, IL-8, TNF-α, and IL-1β in MIO-M1 cells (Fig. 4A). The mRNA levels of GAPDH (internal control) remained largely unchanged in both control and stimulated cells. The semi-quantitative analysis by measuring PCR product band intensity revealed that the highest induction was observed for TNF-α and IL-8 followed by IL-6. The mRNA levels of IL-1β were significantly increased by TLR2 (Pam2) and TLR4 (LPS) agonist compared to other ligands (Fig. 4B). The protein levels of induced cytokines/chemokines were assessed by ELISA. Significantly increased amounts of TNF-α, IL-1β, IL-6, and IL-8 accumulated in the culture media of stimulated MIO-M1 cells for 8 h (Fig. 5C). Consistent with RT-PCR data, Pam2 and LPS induced the maximum secretion of IL-1β.

Previously, we showed that Muller glia secrete proinflammatory mediators in response to *S. aureus*, a Gram-positive bacterium challenge [18]. To assess, whether Muller glia can also respond to other pathogens, we stimulated the MIO-M1 cells to *Pseudomonas aeruginosa*, a Gram-negative bacterium and *Candida albicans*, a fungal pathogen. As shown in the Figure 5, similar to *S. aureus*, Muller glia expressed (Fig. 5A) and secreted increased levels of TNF-α (Fig. 5B) and IL-6 (Fig. 5C) in response to *P. aeruginosa* and *C. albicans*. Taken together, these results indicate that the Muller glia is responsive to different TLR agonists and pathogens via the expression and production of proinflammatory cytokines and chemokines.

### The primary mouse Muller glia expresses functional TLRs

**Figure 6:**
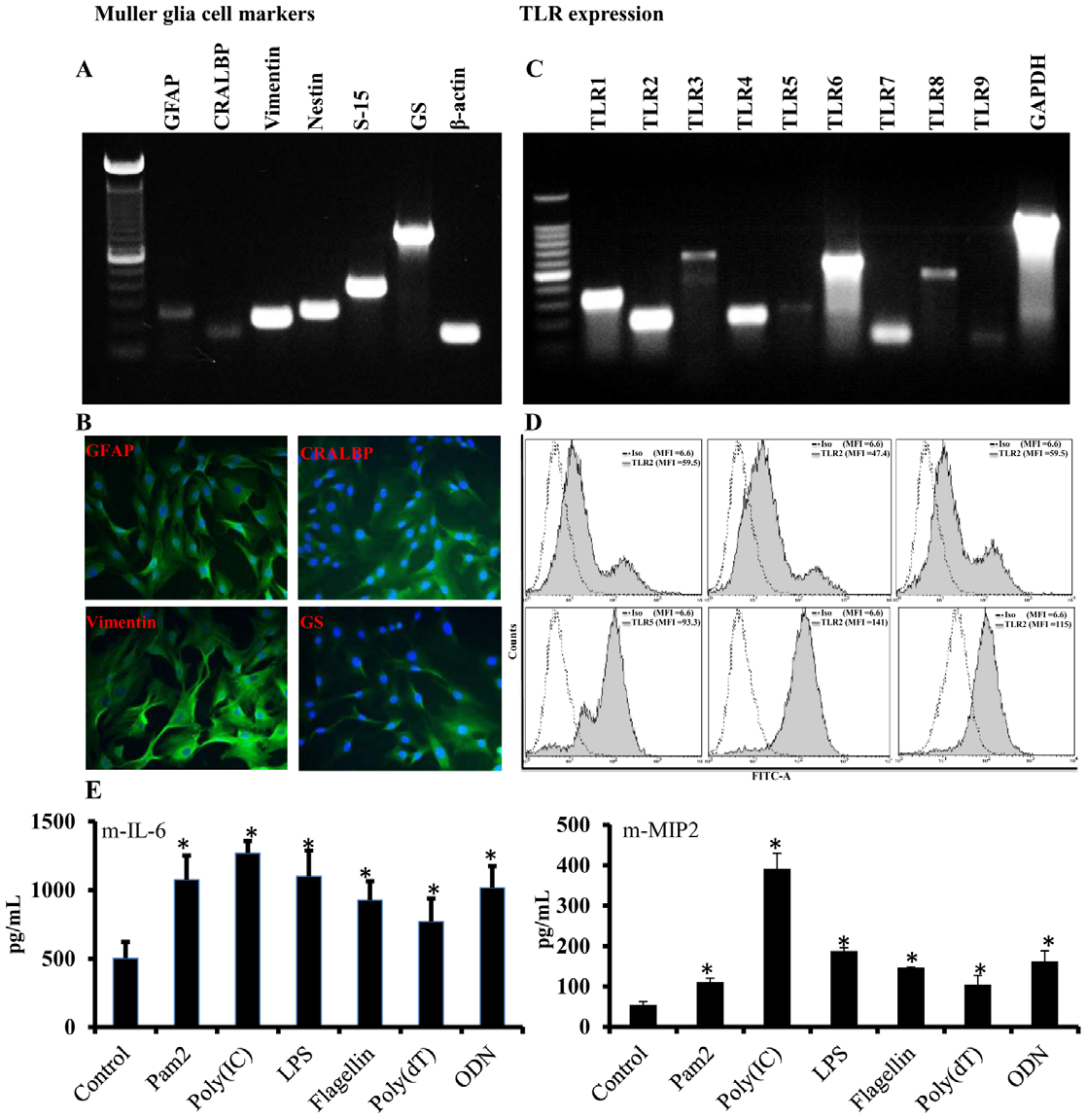
Primary retinal Muller glia express TLRs and are responsive to TLR agonist challenge. The cultured primary retinal Muller glia isolated from neonatal pups of C57BL/6 mice (see methods for detail) were analyzed for the expression of mRNAs encoding Muller cells markers using RT-PCR (**A**) or for protein expression using immunostaining (**B**). The expression of TLRs 1–9 was assessed by RT-PCR (**C**) and flowcytometry (**D**). To determine whether the expressed TLRs are functional, primary Muller glia were stimulated with indicated TLR agonists and secretion of mouse IL-6 and MIP-2 was quantitated by ELISA (**E**). The indicated statistical differences are comparisons of unstimulated (control) vs TLR agonist stimulated cells: *p<0.05 (one-way ANOVA).

Although both *in vivo* (mouse retina) and *in vitro* (MIO-M1) approaches confirmed the expression of TLRs in Muller glia, we further extended these observations in primary cultured mouse Muller glia. To assess the identity of primary Muller glia, we first used the RT-PCR and found that they express mRNA for Muller cell markers, glial fibrillary acidic protein (GFAP), cellular retinaldehyde binding protein (CRALBP), vimentin, nestin, S-15 and glutamine synthetase (GS). The expression levels of GFAP and CRALBP were lower compared to others (Fig. 6A). Furthermore, the expression of GFAP, CRALBP, vimentin and GS at protein levels was confirmed by immunostaining (Fig. 6B). Next, we assessed the TLR expression and observed the mRNA expression of the mouse TLR1 to 9. Among all the TLRs, the mRNA expression of TLR3, 5, and 9 was lower (Fig. 6C). The expression of selected TLRs at protein levels was assessed by flowcytometry (Fig. 6D). To test whether the expressed TLRs are functional, primary Muller glia were stimulated with various TLR agonists and production of inflammatory mediators was assessed by ELISA. Similar, to MIO-M1 cells (Fig. 4), primary Muller glia were also found responsive to TLR ligand challenge, as increased accumulation of mouse IL-6 and MIP-2 was detected in the culture media of stimulated cells (Fig. 6E). Therefore, these findings provided confidence in documenting TLR expression and function in the Muller glia.

## Discussion

The limited immune surveillance of the retina makes it crucial that resident cells be able to quickly recognize and respond to invading pathogens in case of infectious endophthalmitis. We hypothesized that Muller glia provides retinal innate defense in endophthalmitis via the action of TLR signaling and recently reported the expression of functional TLR2 [18]. Infectious endophthalmitis can be caused by a wide variety of microorganisms ranging from bacteria to fungi and each pathogen expresses distinct PAMPs which are recognized by different TLRs. Therefore, it is important to study Muller glial TLR expression profile and their responsiveness to known TLR agonists. To our knowledge, this is the first report describing the expression and function of all known human TLR1–10 in retinal Muller glia. In this study, we show that following stimulation with respective ligands, Muller glia express increased levels of various TLRs and triggered the activation of NF-κB, p38 and Erk MAPKs signaling. Moreover, in response to TLR ligands and live pathogens challenge, Muller glia produced various inflammatory cytokines and chemokines, indicating the functionality of expressed TLRs. Taken together, our data suggests that retinal Muller glia possess the ability to recognize and respond to diverse infectious stimuli, implicating their role in retinal innate immunity.

The discovery of TLRs, which have come to occupy the center stage in innate immunity against invading pathogens, represent a breakthrough in understanding host-pathogen interactions [25]. An increasing number of studies had shown that TLRs are expressed by a variety of tissues and cells in the eye [10], [26]. However, whether retinal Muller glia express various TLRs is not known. We first tested our hypothesis by assessing TLR expression in the mouse retina and showed that Muller glia were immunoreactive to TLR2, 3, 4, 5, 7, and 9 antibodies. However, due to the limitation of available antibodies and their affinities, we could not perform the immunohistochemistry of all TLRs in mouse tissue. Instead, we utilized the available human retinal Muller glia cell line (MIO-MI1) and primary mouse retinal Muller glia for in vitro experiments. Our studies revealed that they express a large repertoire of TLR molecules comprised of TLR1–10 and identified a number of interesting aspects of TLR biology in these cells. The expression of a large set of TLRs suggests that Muller glia have the ability to respond to many microbes that could be encountered in infectious endophthalmitis. Unlike the ocular surface, which is constantly exposed to environmental stimuli including the pathogens, the intraocular compartment of the eye is sterile. Hence, the sensitivity of Muller glia to TLR agonists may be regulated by the level of specific TLR expression. To this end, our western blot and flowcytomerty data showed that in response to TLR ligand stimulation, Muller glia increased the TLR expression levels. This up-regulation of TLRs may enhance the sensitivity of Muller glia to recognize microbes in infectious endophthalmitis.

Since all TLRs were found to be expressed in Muller glia, for which specific ligands were available, we accessed their functional activity upon stimulation with the corresponding ligand. TLR-2 recognizes a variety of microbial products, such as peptidoglycan (PGN) and lipoteichoic acid (LTA) from Gram-positive bacteria, bacterial lipoproteins [27] and zymosan, a yeast cell wall component [28]. Its wide range of ligand specificity may be accountable for its ability to form heterodimers with TLR-1 [29] and TLR-6 [30], [31]. For example, TLR2/6 heterodimer recognizes triacylated lipopeptides, whereas the TLR2/6 heterodimer recognizes diacylated lipopeptides from bacteria. In our recent study, we showed that TLR2 plays an important role in mediating Muller glia innate response to *S. aureus*. We showed that TLR2 activation not only induced the production of inflammatory mediators, but also increased the production of antimicrobial peptides such as LL-37. Moreover, the culture media from TLR2-activated Muller glia possess strong antibacterial activity, suggesting the potential involvement of antimicrobial peptides [18]. In this study, instead of Pam3Cys, a triacylated lipopeptide, we tested the response of Muller glia by using Pam2Cys, which is a diacylated lipopeptide. Taken together, our results indicate that Muller glia express functional TLR1 and 6 and has the potential to recognize both di and tri acylated lipopeptide, mainly from Gram-positive and Gram-negative bacteria respectively. In addition to Gram-positive bacteria, some previous studies have suggested the role of TLR2 in recognition of *C. albicans* [32]. Thus, the responsiveness of Muller glia to *C. albicans* does imply the potential involvement of TLR2.

Lipopolysaccharide (LPS), a major virulence factor present on the cell wall of Gram-negative bacteria is recognized by TLR4, the first mammalian TLR to be characterized [33]. TLR4 signaling requires a number of accessory proteins to initiate a signal. A key component of the TLR4 receptor cluster is MD-2, which binds to the extracellular domain of TLR4 and is essential for LPS recognition [34]. Another important TLR4 accessory protein is CD14, which upon binding to LPS, facilitates the transfer of LPS to the TLR4/MD-2 complex. Studies have demonstrated that this is particularly important in facilitating TLR4 responsiveness to very low levels (<1 ng/ml) of LPS [35]. In addition, CD14 has also been implicated in TLR-2 signaling, and studies have demonstrated that CD14 can bind to lipoproteins [36] and potentiate the activation of NF-κB following TLR2/1 ligation[37]. The expression of TLR4, MD2, and CD14 in Muller glia suggests the existence of functional TLR4 machinery, which was strengthened by our data that showed that these cells are responsive to LPS challenge and *P. aeruginosa*, a Gram-negative bacterium.

In addition to TLR2 and 4, the other receptors involved in bacterial recognition are TLR5 and 9. TLR5 mediates the recognition of flagellin, a flagellum component, present on many motile bacteria [38]. We also previously reported the critical role of TLR5 in corneal innate defense against *Pseudomonas aeruginosa* keratitis [39], [40]. Here, we show that stimulation of Muller glia with either flagellin or live *P. aeruginosa* induces the production of inflammatory mediators. Thus, TLR5 expression on Muller glial cells may be critical in sensing the invasion of flagellated bacteria into the vitreous cavity. TLR3, 7, and 9 belong to the nucleic acid–sensing TLRs family, and they localize to various intracellular compartments. TLR9 recognizes unmethylated 2′-deoxyribo (cytidine-phosphate-guanosine) (CpG) DNA motifs that are frequently present in the bacterial and viral genome but are rare in mammalian cells. TLR3 recognizes dsRNA whereas TLR7-mediates recognition of ssRNA, and their activation initiates antiviral responses. The expression of TLR3, 7, and 9 in Muller glia and their activation with respective ligands implicates the role of Muller glia in antiviral innate defense, potentially in viral retinitis in AIDS patients [41]. Overall, our data suggests that Muller glia possesses the ability to respond to diverse pathogen associated molecular patterns (PAMPs) present on bacterial, fungal and viral pathogens. Although, we recently reported that siRNA-mediated knockdown of TLR2 attenuated the responsiveness of Muller glia towards *S. aureus* [42], further studies are warranted to ascertain the specific roles of TLR(s) in recognizing Gram-negative bacteria and fungi. Moreover, in addition to TLRs, we cannot exclude the possibility that other pattern recognition receptor families, e.g. NOD-like receptors NLRs and retinoic acid-inducible gene-I-like receptors (RLRs), may also play a role in pathogen recognition by Muller glia.

Although the recognition of different ligands by specific TLRs leads to activation of an intracellular signaling cascade in a MyD88 dependent or independent fashion, all TLRs share NF-κB signal transduction pathways to regulate many key proteins participating in inflammation [10]. In retinal Muller glia, activation of all TLRs by their respective ligands appears to trigger a classical response characterized by a nuclear translocation of the NF-κB p65 subunit, as evaluated by indirect immunofluorescence. In addition to NF-κB activation, TLR ligands also induce the activation of other MAPKs, such as p38 and Erk, and these signaling molecules are known to contribute towards the inflammatory response in cells [17]. Our data, that TLR agonist induces the activation of p38, and Erk suggests their involvement in Muller glial innate response to infectious stimuli.

**Figure 7:**
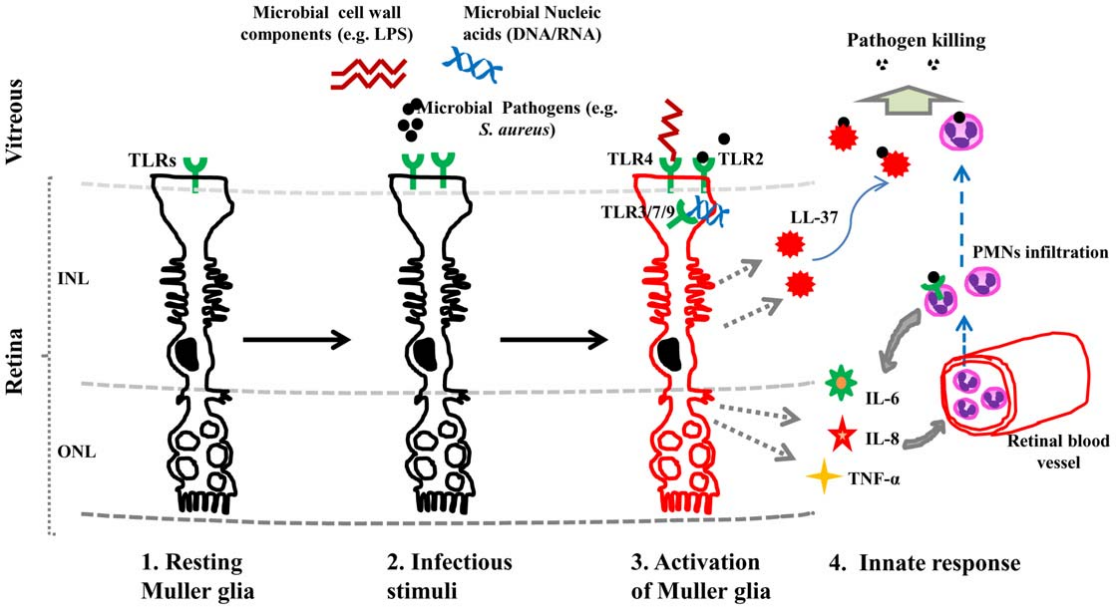
Summary of retinal Muller glial innate response to infectious stimuli. (***1***) Retinal Muller cells are specialized glial cells, which span through the entire thickness of the retina. Under normal conditions, they express Toll-like receptors (TLRs) at the basal level. (***2***) During trauma or intraocular surgeries, the microbial pathogens or their products (e.g. LPS) gain access to vitreous cavity, where they activate Muller glial expressed TLRs. (***3***) Binding of TLRs by its specific ligand (e.g. LPS for TLR4, *S. aureus* for TLR2 or bacterial CpG DNA by TLR9) leads to up-regulation of TLRs and activates pro-inflammatory signaling cascade, leading to secretion of various inflammatory cytokines (IL-6, TNF-α), chemokines (IL-8), and antimicrobial peptides (LL-37). (***4***) Muller glia released chemoattractants, such as IL-8 diffuse across retinal blood vessels and drive PMN infiltration into the retina. While initiating phagocytosis to eliminate the invading microbial pathogens, infiltrated PMNs also release chemoattractants through TLR activation by localized bacterial products to perpetuate PMN infiltration. The TLR-mediated secretion of antimicrobial peptides (LL-37) can directly kill the invading pathogens. Thus, the Muller glia possesses the ability to sense infectious stimuli and initiate innate responses via the action of TLR-signaling.

In conclusion, our findings show that Muller glia express multiple TLRs, and TLR ligand treatments enhanced the expression of respective TLRs, thus providing a mechanism to potentate Muller glia activation prior to the infiltration of leukocytes from the peripheral circulation (Fig. 7). Thus, elucidation of the cellular pathways utilized by Muller glia in response to the infectious stimuli may provide novel means to manipulate the intraocular immune response in endophthalmitis.

## Materials and Methods

### Ethical Statement

Mice were treated in compliance with the ARVO statement for the Use of Animals in Ophthalmic and Vision Research, and all procedures were approved by the Institutional Animal Care and Use Committee of Wayne State University (protocol A- 09-07-10).

### Pathogens, TLR agonists, and antibodies

*S. aureus* (strain RN6390) and *P. aeruginosa* (ATCC 19660) were maintained in tryptic soy broth (TSB; Sigma-Aldrich, St. Louis, MO) whereas C. albicans (strain SC5314) was cultured on YPD agar (Sigma-Aldrich, St. Louis, MO). Bacterial lipopeptide Pam2Cys-Ser-(Lys)_4_ hydrochloride (Pam2Cys, TLR2 agonist), polyI:C (TLR3 agonist), Lipoploysacchride (LPS, TLR4 agonist), flagellin (TLR5 agonist), PolydT (TLR7/8 agonist), and ODN (TLR9 agonist) were purchased from Invivogen (San Diego, CA). Anti-phospho-p38 MAPK mAb, anti-p38 antibody, anti-phospho-IκB-α, anti-IκB-α, anti-TLR2, and HSP-90 antibodies were purchased from Cell Signaling Technology (Beverly, MA). Antibodies against other TLRs were obtained from Santa Cruz Biotechnology Inc (CA, USA). A mouse monoclonal anti-β-actin antibody was purchased from Sigma-Aldrich (St. Louis, MO). Secondary horseradish peroxidase (HRP)-conjugated anti-mouse or anti-rabbit IgG antibodies were purchased from Bio-Rad (Hercules, CA).

### Muller glia cell line and primary culture

Spontaneously immortalized Human Muller Cell Line (MIO-M1), kindly provided by Dr. G. Astrid Limb, were maintained in DMEM (Hyclone, South Logan, UT) supplemented with Glutamax (10%), FBS (10%), and antibiotic cocktail in a humidified 5% CO_2_ incubator at 37°C. At the time of treatment, the culture medium was replaced with antibiotic-free and growth-factor-free fresh DMEM without phenol red (Hyclone, South Logan). At the indicated times (as shown in figures), the cells were processed for RNA extraction and protein preparation, whereas the conditioned media were collected for cytokine assays.

Mouse primary retinal Muller glia were cultured from 2–3 day old neonatal pups of C57BL/6 mice. Animals were euthanized and their eyes enucleated. The globes were dissected and rinsed with Hanks Balanced Salt Solution (HBSS), transferred into 2% dispase, and placed in a 5% CO2 incubator at 37°C for 1 h. Dispase activity was neutralized by washing the globes with low glucose DMEM (Hyclone, South Logan, UT) supplemented with 10% FBS (BioAbchem, Ladson, SC). The anterior segment and vitreous were excised, and the RPE layer was removed. The retinas were transferred into DMEM containing 10% FBS and triturated several times with a pipette. The dissociated cells were transferred into 75 cm2 flasks and left to grow at 37°C for 3–4 days. After the mixed culture had grown confluent, the flasks were subjected to mechanical shaking to remove loosely attached cells primarily microglia. The cells, firmly attached to bottom, consisting 90% Muller glia, were then cultured in 100 mm dishes at a low density. Muller glia divided over the next two weeks to form individual colonies of adherent cells. Individual cell clusters comprised solely of Muller glia were trypsinized inside of a colony cylinder and cultured in a new 75 cm2 flask. The purity of Muller glia in this resulting culture exceeded >98%.

### RNA extraction and RT-PCR analysis

Total RNA was isolated from MIO-M1 cells using the TRIzol solution, following the manufacturer's instructions (Invitrogen, Carlsbad, CA) and 2 µg of total RNA was reverse-transcribed with a first-strand synthesis system for RT-PCR (SuperScript; Invitrogen). cDNA was amplified using human (Table 1) or mouse (Table 2) specific primers. The PCR products and internal control GAPDH or β-actin was subjected to electrophoresis on 1.2 % agarose gels containing ethidium bromide. Stained gels were captured using a digital camera (EDAS 290 system, Eastman Kodak, Rochester, NY), and band intensity was quantified using Image J analysis software (NIH).

**Table 1:** List of human primers used for RT-PCR analysis.

Gene	Primer sequence	Product Size (bp)	Tm (°C)
TLR1	F: CTATACACCAAGTTGTCAGCR: GTCTCCAACTCAGTAAGGTG	219	55
TLR2	F: GTGGCCAGCAGGTTCAGGATGR: AGGACTTTATCGCAGCTCTCAG	641	55
TLR3	F: GATCTGTCTCATAATGGCTTGR: GACAGATTCCGAATGCTTGTG	304	55
TLR4	F: TGGATACGTTTCCTTATAAGR: GAAATGGAGGCACCCCTTC	506	55
TLR5	F: TAGCTCCTAATCTGATGR: CCATGTGAAGTCTTTGCTGC	355	60
TLR6	F: CCTCAACCACATAGAAACGACR: CACCACTATACTCTCAACCCAA	531	55
TLR7	F: TCTACCTGGGCCAAAACTGTTR: GGCACATGCTGAAGAGAGTTA	388	55
TLR8	F: GCCAGCGAGTCTCACTGAACTR: GCCAGGGCAGCCAACATA	443	55
TLR9	F: GTCCCCACTTCTCCATGR: GGCACAGTCATGATGTTGTTG	259	55
TLR10	F: TTATGACAGCAGAGGGTGATGCR: CTGGAGTTGAAAAAGGAGGTTATAGG	151	55
CD14	F: CTCAACCTAGAGCCGTTTCTR: CAGGATTGTCAGACAGGTCT	426	55
MD2	F: TATTGGGTCTGCAACTCATR: CTCCCAGAAATAGCTTCAAC	359	55
TRIF	F: CTATAACTTTGTGATCCTCR: GGTGTTGGCCACCTTCCT	399	55
TRAM	F: GGCAAAGGGGATCCTTTCTCGGGGAAR: GCACAGTGTGGATCCAAGTCCAGGATA	505	60
TRAF-6	F: TTCATAGTTTGAGCGTTATACCCGACR: CAGACTGATCAAGAATTGTAAGGCGTA	485	55
MyD88	F: TGCTGGAGCTGGGACCCAGCATTGAGGAGGAR: TCAGACACACACAACTTCAGTCGATAG	503	55
TNF-α	F: GAAAGCATGATCCGGGACGTGR: GATGGCAGAGAGGAGGTTGAC	510	55
IL-1β	F: AAACAGATGAAGTGCTCCTTCCAGGR: TGGAGAACACCACTTGTTGCTCCA	390	55
IL-6	F: CTCCTTCTCCACAAGCGCCTTCR: GCGCAGAATGAGATGAGTTGTC	583	55
IL-8	F: GCAGTTTTGCCAAGGAGTGCTAR: GCATCTGGCAACCCTACAACAAG	347	55
GAPDH	F: CACCACCAACTGCTTAGCACR: CCCTGTTGCTGTAGCCAAAT	515	55

**Table 2:** List of mouse primers used for RT-PCR analysis.

Gene	Primer sequence	Product Size (bp)	Tm (°C)
TLR1	F: GTTGTCACTGATGTCTTCAGCR: CTGTACCTTAGAGAATTCTG	319	55
TLR2	F: TGCTTTCCTGCTGGAGATTTR: TGTAACGCAACAGCTTCAGG	196	55
TLR3	F: CAGTTCAGAAAGAACGGR: AGCCTTATACCATAAAAGC	614	55
TLR4	F: TTGTCTTCTGCACGAACCTGR: GGCAACGCAAGGATTTTATT	311	55
TLR5	F: GGTGGTGGTGGGATCGCTGTCR: CCAGGGAAGGACCCCCAGCG	295	55
TLR6	F: AGTGCTGCCAAGTTCCGACAR: AGCAAACACCGAGTATAGCG	527	55
TLR7	F: TCTTACCCTTACCATCAACCACAR: CCCCAGTAGAACAGGTACACA	111	55
TLR8	F: CTTTCCAGCACTTCCCTCAGR: GAAGACGATTTCGCCAAGAG	459	55
TLR9	F: TGAAGTCTGTACCCCGTTTCTR: GTGGACGAAGTCGGAGTTGT	106	55
GFAP	F: CACGAACGAGTCCCTAGAGCR: ATGGTGATGCGGTTTTCTTC	234	60
CRALBP	F: TACCCTGGTGTCCTTTCCAGR: GGTTTCCTCATTTTCCAGCA	150	63
Vimentin	F: ATGCTTCTCTGGCACGTCTTR: AGCCACGCTTTCATACTGCT	206	64
Nestin	F: AACTGGCACACCTCAAGATGTR: TCAAGGGTATTAGGCAAGGGG	235	60
S-15	F: TTCCGCAAGTTCACCTACCR: CGGGCCGGCCATGCTTTACG	361	60
GS	F: CCACCTCAGCAAGTTCCCACTTR: TCAGACCATTCTCCTCCCGCAT	797	55
β-actin	F: CTTCTTTGCAGCTCCTTCGTR: GTGCCAGATCTTCTCCATGT	310	55
GAPDH	F: TCATTGACCTCAACTACATGGTR: CTAAGCAGT TGGTGGTGCAG	442	55

### Flow cytometry analysis

MIO-M1 cells treated with or without ligands were dispensed in eppendorf tubes and centrifuged at 100 *g* at 4°C for 5 min. Cells were then resuspended in PBS containing 1% bovine serum albumin (BSA). The cells were then blocked in 10% serum for 30 minutes at room temperature. The cells were then washed, resuspended in PBS containing anti-TLR2, 3, 4, 5,7, and 9 antibodies (1∶200 dilution) or isotype matched IgG (SantaCruz Biotech, Santa Cruz, CA) (1∶20 dilution) and were incubated overnight at 4°C. Cells were washed and incubated with the corresponding secondary FITC-conjugated antibody (Invitrogen) (1∶200 dilution) for 30 min. A BD LSR II flow cytometer (Immunocytometry Systems, Becton and Dickinson, San Jose, CA) was used for cytometric analysis.

### Western and Dot Blot analysis

MIO-M1 cell lysates were prepared with radioimmunoprecipitation assay (RIPA) buffer [150 mM NaCl, 100 mM Tris-HCl, pH 7.5, 1% deoxycholate, 0.1% sodium dodecyl sulphate (SDS), 1% Triton X-100, 50 mM NaF, 100 mM sodium pyrophosphate, 3.5 mM sodium orthovanadate, proteinase inhibitor cocktails (Sigma-Aldrich, St. Louis, MO), and 0.1mM phenylmethylsulphonyl fluoride (PMSF)], and the protein concentration was determined using the BCA assay (micro BCA; Pierce, Rockford, IL). Proteins (30–40 µg/well) were separated by sodium dodecyl sulphate–polyacrylamide gel electrophoresis (SDS-PAGE) in Tris/glycine/SDS buffer (25 mM Tris, 250 mM glycine and 0.1% SDS) and electro-blotted onto nitrocellulose transfer membranes (Bio-Rad Laboratories, Hercules, CA). After blocking for 1–2 h in phosphate-buffered saline with Tween-20 (PBST) (20 mM Tris-HCl, 150 mM NaCl and 0.5% Tween) containing 5% non-fat milk, the blots were probed overnight at 4°C with the desired antibodies as described by the manufacturer (Cell Signaling Technology; Sigma Aldrich). NF-κB activation was determined in terms of inhibitory IκB-α phosphorylation and degradation using anti-IκB-α and anti phospho-IκB-α antibodies. After washing three times in PBST, membranes were incubated with secondary horseradish peroxidase (HRP)-conjugated anti-mouse or anti-rabbit IgG antibodies (Bio-Rad) for 1 h. After washing with PBST four times for 10 min each, proteins were visualized with Super signal reagents from Pierce (Rockford, IL).

### Enzyme-linked immunosorbent assay (ELISA) for cytokine analysis

MIO-M1 cells were plated (5×10^6^ cells per well) in six-well plates. After growth factor starvation, cells were challenged with different TLR specific agonists viz Pam2 (TLR2/6), PolyI:C (TLR3), LPS (TLR4), flagellin (TLR5), poly(dT) (TLR7/8) and ODN (TLR9) for indicated time periods, and culture media were collected for measurement of human TNF-α, IL-1β, IL-6, and IL-8 by ELISA. The cells were lysed, and protein concentration was determined. ELISAs were performed according to the manufacturer's instructions (R&D Systems, Minneapolis, MN). The amount of cytokines in the culture media was expressed as picograms per mg of cell lysate.

### Immunoflourescence staining

Retinal cryo-sections were rinsed in PBS and blocked for 1 h in blocking buffer [10% (vol./vol.) normal goat serum, 0.3% (vol./vol.) triton X-100 in PBS] at room temperature. The slides were then incubated overnight with anti-TLR2, 3, 4, 5, 7 and 9 (Santa Cruz) or anti vimentin (Sigma Aldrich) antibody at 4°C. Following removal of the primary antibodies, slides were extensively washed and incubated for 1 h in fluorescent-conjugated secondary antibodies (FITC) at room temperature. After several washing steps in PBS, the slides were mounted in Vectashield anti-fade mounting medium (Vector Laboratories, Burlingame, CA, USA) and visualized using a confocal system (Leica Microsystems, Wetzlar, Germany).

For *in vitro* staining, MIO-M1 cells cultured on glass chamber slides (Fisher Scientific, Rochester, NY) were stimulated with different TLR ligands for indicated time periods. The cells were washed three times with phosphate-buffered saline (PBS) and then fixed for 15 min in PBS with 4% paraformaldehyde. After washing with gentle shaking, the cells were permeabilized for 5 min with ice-cold methanol and washed. The fixed cells were then blocked in 5% (wt/vol) serum for 1 h at room temperature followed by incubation with TLR2, 3, 4, 5, 7 and 9 (Santa Cruz, CA) (1∶200 dilution) antibodies overnight at 4°C. In control experiments, cells were incubated with non-immune IgG as isotype controls (Santa Cruz). Following removal of the primary antibodies, the cells were then extensively washed with PBS and incubated for 1 h with specific fluorescein isothiocyanate (FITC/Rhodamine)-conjugated secondary antibodies (1: 200 dilution) at room temperature. Finally, the cells were washed extensively with PBS, and the slides were mounted in Vectashield anti-fade mounting medium (Vector Laboratories) and visualized using a confocal system (Leica Microsystems).

### Statistics

All values were expressed as mean ± standard deviation (SD). The statistical analysis was performed using an unpaired *t*-test for comparison of two groups whereas one-way analysis of variance (ANOVA) was performed for multiple group comparisons (Prism; GraphPad Software). *p* values less than 0.05 were considered significant.
