# Correction: Retinal Muller Glia Initiate Innate Response to Infectious Stimuli via Toll-Like Receptor Signaling

**DOI:** 10.1371/annotation/951b4189-ee76-4a19-98f4-504f4355c45c

**Published:** 2012-02-29

**Authors:** Ashok Kumar, Nazeem Shamsuddin

The equal contributions designation was omitted. The two authors of the article contributed equally to the work. 

